# Effects of on-farm and traditional hatching on welfare, health, and performance of broiler chickens

**DOI:** 10.1016/j.psj.2020.06.052

**Published:** 2020-07-08

**Authors:** Ingrid C. de Jong, Theo van Hattum, Johan W. van Riel, Kris De Baere, Ine Kempen, Sofie Cardinaels, Henk Gunnink

**Affiliations:** ∗ Wageningen Livestock Research, 6700 AH Wageningen, The Netherlands; † Experimental Poultry Centre, Province of Antwerp, 2440 Geel, Belgium

**Keywords:** broiler, health, on-farm hatching, production, welfare

## Abstract

In on-farm hatching systems, eggs that have been incubated for 18 D are transported to the broiler farm. After hatching around day 21, the chicks have immediate access to feed and water. By contrast, traditionally hatched chicks are in early life exposed to dust and pathogens in the hatcher, handling procedures, and transport and remain without feed and water until they have arrived on the farm 1 to 3 D after hatching. We compared welfare and performance of on-farm hatched (**OH**) and traditionally hatched control (**C**) Ross 308 broiler chickens from day 0 to 40, housed under semicommercial conditions. The experiment included 3 production cycles in 4 rooms, with each room containing 1 OH and 1 C pen with 1,150 chickens in each pen. Per cycle, C and OH chicks were from the same batch of eggs of 1 parent stock flock. Day-old chick quality was worse for OH than C chickens (hock and navel score; *P* < 0.05). On-farm hatched chickens were heavier than C chickens until day 21 of age (*P* < 0.05). Total mortality was significantly lower in OH compared with C pens (*P* < 0.05). A tendency for lower footpad dermatitis scores was found in OH pens compared with C pens (*P* < 0.10), probably because of the dryer litter in OH than C pens (*P* < 0.05). No differences between treatments were found in gait, hock burn, cleanliness, and injury scores, and no or only minor, short lasting differences were found in pathology and intestinal histology. In conclusion, the present study showed that on-farm hatching may be beneficial for broiler welfare, as it reduced total mortality and resulted in dryer litter which is known to be beneficial for reducing footpad dermatitis.

## Introduction

Broiler chickens traditionally hatch in the hatchery, and after removal of second-grade chicks and vaccination, they are transported to the broiler farm and placed as day-old chicks, where they receive their first feed and water. As a consequence, the posthatch feed and water deprivation period may last up to 72 h, depending on the length of the hatch-window and time needed for selection and transport (Careghi et al., 2005; Willemsen et al., 2010). We have previously shown, however, that a posthatch feed deprivation of more than 36 h may result in increased mortality and impaired growth and feed conversion as compared with providing chickens their first feed immediately posthatch (de Jong et al., 2017). Moreover, early posthatch feed deprivation may impair gut development and broiler health, although this merits further study as not all studies have shown significant negative effects (de Jong et al., 2017). In addition to this posthatch feed deprivation, chicks in conventional hatching systems are exposed to disinfection, high dust, and pathogen loads (de Gouw et al., 2017), high noise levels, continuous darkness (Archer and Mench, 2014), handling (vaccination, selection) (Knowles et al., 2004; Hedlund et al., 2019), and transport (Mitchell, 2009; Jacobs et al., 2017; Hollemans et al., 2018). These are major stressors (Mitchell, 2009; de Gouw et al., 2017; Hedlund et al., 2019) that could have long-term consequences on the health and welfare of the chickens (Elfwing et al., 2015; Ericsson et al., 2016; Hedlund et al., 2019). For example, Hedlund et al. (2019) found that standard hatchery procedures were stressful for laying hen chickens, resulting in long-term changes in behavior and stress reactivity.

To improve the early postnatal conditions and as a result health, welfare, and performance of broiler chickens, systems have been developed where the chicks hatch on-farm. Eighteen days incubated eggs are transported to the farm and placed in racks, boxes, or in the litter where they hatch in the house providing the chicks with immediate access to feed and water without exposure to aforementioned stressors (transport, handling, noise, darkness, dust, and pathogens) at the hatchery. However, until now, on-farm hatching is practiced at a rather limited scale and almost only in the North-Western part of Europe and Russia (Van Wagenberg, Vencomatic Group, personal communication), maybe because of the financial investment and skill requirements of farmers managing the hatching process or because the benefits of on-farm hatching with respect to production, health, and welfare are yet unclear. For example, only about 4% of the broiler chickens produced in The Netherlands hatched on-farm in 2019 (Stuurgroep Pluimveesector Circulair, 2019).

There is indeed only limited scientific evidence about the effects of these on-farm hatching systems on broiler health, welfare, and performance. In a recent study, we (de Jong et al., 2019) compared performance and welfare of broiler flocks that hatched conventionally or on-farm under commercial conditions. We found that on-farm hatched (**OH**) flocks had less footpad dermatitis and a better litter quality than conventionally hatched flocks, but we did not find any long-term effects on technical performance, health or intestinal development. However, the study was conducted on commercial farms, and although we controlled for parent stock and used paired identical houses on a farm for both treatments, individual farmer management may have had an effect on the outcomes of the study. As concluded in de Jong et al. (2019), a follow-up study under more controlled conditions was called for.

Therefore, the aim of the present study was to compare performance, health, and welfare of broiler chickens that hatched on-farm as compared with broiler chickens that were traditionally hatched, under controlled conditions at an experimental farm. Based on scientific evidence about positive effects of immediate posthatch feeding on mortality and performance (de Jong et al., 2017) and long-term effects of stress around hatching on laying hen stress sensitivity (Hedlund et al., 2019), we hypothesized that the reduction in stressors around hatching of broiler chickens with on-farm hatching would lead to improved technical performance, health, and welfare of broiler chickens until slaughter age for OH chickens as compared with chickens that hatched in the hatchery. Because both environmental conditions at hatching and early feeding may affect day-old chick quality, which may in turn affect further performance (Tona et al., 2005; Willemsen et al., 2010; de Jong et al., 2017), day-old chick quality indicators were included in the present experiment.

## Materials and methods

### Study Design and Housing

The project approval was received on June 10, 2015 by the Central Commission on Animal Experiments (licence number AVD40100201563), and the experiment was approved by the Institutional Animal Care and Use Committee on August 12, 2016.

The experiment was carried out at the Experimental Poultry Center in Geel, Belgium, during 3 successive production cycles between August 2016 and January 2017. Two treatments were applied: traditional hatching at the hatchery (Control, **C**) and OH. Control broilers were hatched at a commercial hatchery (Spoormans, Arendonk, Belgium) according to standard commercial procedures. Chicks were collected from the incubators when the majority had hatched, followed by standard hatchery procedures such as removal of second-grade chicks (but without receiving any vaccinations), and the chicks were transported to the experimental farm at Day (**D**) 0. For the OH treatment groups, 18 D incubated eggs from the same batch of eggs and parent stock as the C treatment were transported to the broiler farm, placed in the X-treck system (see below), and hatched in the broiler house. Both C and OH eggs were candled at embryonic day (**E**) 18 (before transport of OH eggs to the farm). For each of the 3 production cycles in the experiment, at E18 of incubation trays were alternately assigned to the C or OH treatment by the hatchery. In each production cycle, 4 OH pens were equipped with the X-treck systems, and 4 C pens were stocked with traditionally hatched chicks as described below.

The X-treck system (Vencomatic, Eersel, The Netherlands) consists of setter trays that are placed on a suspended rail system 14 to17 cm above a polypropylene belt covered with substrate, which is placed 33 cm above the floor. After on-farm hatching, chicks fall on the belt. After drying on the belt, they move to the edge of the belt and fall on the litter, where feed and water is provided. Trays with egg shells and nonhatched eggs are removed from the house at D0, and the system can be lifted to the ceiling after use.

In each production cycle, the same 4 identical rooms were used. Each room was equipped with central heating and its own climate control system. Equal climate settings were applied in all rooms and production cycles. The 4 rooms were located next to each other in 1 broiler house and connected by a central corridor from which each room could be entered. Each room contained 2 pens (each pen measuring 6 × 9.4 m), separated by a wire mesh partially covered with a hardboard plate to prevent bird-to-bird contact between pens. One pen per room was assigned to the C treatment, and the other pen to the OH treatment, resulting in 4 replicates per treatment for each of the 3 successive production cycles. The location of C and OH pens relative to the entrance door alternated per room but was equal for the 3 cycles. Pens had their own automated feeders and drinkers, enabling registration of feed and water intake at pen level. Each pen was equipped with 14 feeder pans distributed over the pen and 2 drinker lines with 84 nipples in total. Fresh crushed straw pellets (1.5 kg/m^2^) as litter material were distributed before placement of the eggs, and in the OH pens, containing the X-treck system, a small amount of litter material was distributed over the conveyor belts.

### Animals and Management

In total 27,780 Ross 308 broiler chickens (as hatched) were used. The parent flocks were 41, 35, and 39 wks of age in the first, second, and third production cycle, respectively. The day at which the C chicks arrived from the hatchery was, according to commercial practice, named “D0” for both treatments. Control chicks, 1,150 per pen, arrived from the hatchery and were placed in the morning of D0. In OH pens, the caretakers removed nonhatched eggs and selected second-grade chicks, removed the setter trays, and lifted the rail of the X-treck system to the ceiling in the morning of D0. Nonhatched eggs were shredded, and second-grade chicks were killed by cervical dislocation. After selection in the OH pens on average 1,176, 1,165, and 1,154 day-old chicks were present at D0 in cycle 1, 2, and 3, respectively. The number of eggs placed in the second and third production cycle was adjusted according to the hatching results in the previous cycle to be as close to the aimed number of 1,150 chicks per pen at D0 and thus a final stocking density at slaughter weight of 42 kg/m^2^ according to legislation in all pens.

Continuous light was on from E18 (being the day the eggs were placed in the OH pens) up till D0 to enable the chicks to find food and water after hatching. All chicks received vaccinations for infectious bronchitis and Newcastle disease at D0 (in the broiler house) and D13, and Gumboro vaccination at D19. At D0, C chicks received their spray vaccinations in the boxes before placement which took approximately 15 min. On-farm hatched chicks received their vaccinations by spraying the pen. Feed was provided on chick paper during the first days, starting at E18 for OH pens. Drinking nipples were placed on the litter at D0, and nipple height was increased with age. A standard commercial 4-phase feeding program was applied (Aveve, Merksem, Belgium), and both food and water were provided *ad libitum*. On D0, 1 h of darkness was provided which increased to 6 h of darkness from D6 onward; lights were on from 04:00 to 07:00 h, 08:00 to 20:00 h, and 21:00 to 00:00 h. During the final 3 D before depopulation 1 h of darkness was provided from 00:00 h to 01:00 h. Light intensity was 20 lux at animal height. The environmental temperature decreased from 35°C at D0 to 19°C at D40. For the OH groups, the environmental temperature between E18 and D0 was based on measurements of the egg-shell temperature, recorded on E18 and E19. The preferred egg-shell temperature was 37.8°C. The average environmental temperature from E18 to D0 was 35°C with a relative humidity of 40 to 45%. Thinning was performed once at D33 as a standard commercial procedure by sending 280 broilers from each pen to the slaughterhouse. The remainder of the birds stayed until all pens were depopulated at D40. After 1 wk, which was used for cleaning and disinfection, a new cycle started with the placement of 18-D incubated eggs in the OH pens. It was decided beforehand that no antibiotic treatments would be applied during the study.

### Measurements

#### Day-old Chick Quality and Development

At D0, 6 chicks were randomly selected per pen after removing the second-grade chicks (OH treatment) or from different chick boxes just before placement in the pen (C treatment). These chicks were weighed, killed by decapitation, and scored for navel and hock quality. Navels were scored on a scale from 1 (good) to 3 (worst) as described by Van der Pol et al. (2013). Hocks were also scored on a 3-point scale (1 = no red hocks; 2 = slightly red hocks; 3 = red hocks, skin possibly damaged). Chick length was measured according to Nangsuay et al. (2011). Organs (heart, gizzard plus proventriculus, gut, liver, and yolk sac) were dissected and weighed. Yolk-free body mass was calculated as BW minus yolk sac weight. All organ weights were expressed as percentage of yolk-free body mass. Crops were opened and checked for the presence of feed. Finally, total gut length and intestinal length were measured for each chicken. All measures and scores were performed by 2 pretrained observers.

#### Technical Performance

Technical performance was measured by personnel of the experimental farm. Feed and water intake were recorded at pen level during the whole experimental period. A sample of 50 randomly chosen chickens per pen was weighed at D0, 1, 7, 21, and 29; at D33, all thinned birds (280 chickens per pen) were weighed, and at D40, all remaining birds were weighed at depopulation. Body weights at D0 were taken from a different sample of 50 chicks per pen than for measurements of chick quality and measured 2 to 5 h after placement of the birds. Mortality and culls were recorded daily. Both the FCR corrected to 1,500 g (FCR1500; correction factor 0.01 per 25 g) or to 2,500 g (FCR2500; correction factor 0.01 per 50 g) were calculated from the data, in addition to the net FCR at slaughter age. From the performance data, the European Production Efficiency Factor was calculated: EPEF = {BW gain (g/D) × [100 − mortality (%)]}/(feed conversion × 10).

#### Litter Moisture Level and Litter Quality

Litter moisture level was determined by farm personnel. Litter samples were collected from each pen at D7, 14, 21, 28, 34, and 39 on 3 locations (near the feeders, near the drinkers, and in the middle of the pen) and thoroughly mixed. A total of 200 (D7), 300 (D14), 400 (D21), 600 (D28), 800 (D34), and 1,000 gr (D39) per pen was dried to determine the dry matter percentage. In addition, litter quality was scored by 2 pretrained observers at the same locations in each pen on D4, 8, 14, 21, 28, 35, and 39 by visual inspection and classified on a scale between 0 (completely dry and loose) and 4 (very wet or completely capped with a crust) (Welfare Quality, 2009).

#### Gait, Footpad Dermatitis, Hock Burn, Cleanliness and Injuries

At D21 and D35, a total of 30 broilers per pen were collected randomly in a catching pen for individual gait scoring according to Welfare Quality (2009) on a scale from 0 (perfect locomotion) to 5 (unable to walk). Thereafter, again 30 broilers per pen were randomly collected in a catching pen and inspected for footpad dermatitis, hock burn, cleanliness, and injuries (scratches and wounds). Footpad dermatitis and hock burn were scored on a scale from 0 (no lesions) to 4 (severe lesions on the foot or hock) (Welfare Quality, 2009). Cleanliness was scored by inspection of the belly on a scale between 0 (clean) and 3 (very dirty) (Welfare Quality, 2009). Injuries were scored on a 3-point scale (0: no injuries or a maximum of 3 scratches; 1: single lesion smaller than 2 cm^2^ or more than 3 scratches; 2: at least one lesion larger than 2 cm^2^). All scores were performed by 2 pretrained observers.

#### Dissections and intestinal histology

At weekly intervals, 6 chickens per pen (randomly selected, but including 3 males and 3 females from 4 wk of age onward), were examined by 1 veterinarian to score the intestines for coccidiosis (Johnson and Reid, 1970) and dysbacteriosis (Teirlynck et al., 2011), gross pathology (inspection of organs, such as heart, liver, trachea, air sacs, lungs, kidney, proventriculus, gizzard and bursa, and clinical signs of disease), femoral head necrosis, and tibial dyschondroplasia (TD) for each leg separately. Dysbacteriosis was scored on a scale from 0 (normal gastrointestinal tract) to 10 (most severe dysbacteriosis) (Teirlynck et al., 2011). Coccidiosis was scored on a scale from 0 (no signs) to 4 (severe signs) for *E. acervulina*, *E. maxima,* and *E. tenella* (Johnson and Reid, 1970), and scores per type were added (thus, the sum total could vary between 0 and 12). Femoral head necrosis was assessed by dislocating the femur and scored as follows: 0 = intact femur, 1 = red irritation, and 2 = femur fracture before or as a consequence of dislocation. In addition, total gut length and intestinal, cecal, and colon length separately were determined and expressed as ratio length:BW. Similarly, the proximal growth plate of the tibia was cut open to assign a score for TD (0 = no visual signs of TD; 1 = small cartilage lesion; 2 = large cartilaginous plug in the growth plate).

Only for the second production cycle, tissue samples were taken from the mid-jejunum of the above indicated 6 chickens per pen at D8, D14, and D21. Samples were collected within 30 s after euthanasia of the chickens. The intestines were injected with 1 to 2 ml 4% buffered formalin, and a tissue sample of 1 to 2 cm^2^ was collected from the jejunum and fixed in 4% buffered formalin at room temperature until further analysis for villus height and crypt depth by the Animal Health Service (Deventer, The Netherlands), where the samples were dehydrated and embedded in paraffin. Tissue sections of 2 μm were stained with hematoxylin and eosin. Microscopic images of representative cross-sections of each tissue were captured by a microscope (Olympus BX41) connected to a digital camera (Olympus Dp26) and analyzed using Olympus cellSens Dimension version 1.12 software. Of each jejunal segment, 10 representative and completely paired villus-crypt units were measured. The villus:crypt ratio was determined as the length of the villi divided by the depth of the mucosal crypt region. The average of each villus:crypt ratio and the neutral and acid mucin producing goblet cell characteristics (number and proportion per villus, size, area, and area as proportion of villus area) were calculated and reported per chicken.

### Statistical Analysis

All analyses were performed using GenStat (version 19.1, VSN International). Differences with *P* < 0.05 were considered statistically significant, and 0.05≥P ≤ 0.10 were considered a trend. Because treatments were allocated to individual pens which were similar for all productions cycles, the scores of individual chickens were aggregated over production cycles per pen (for each combination of age or sex, if needed). The normality of the data was checked using residual plots. A natural log transformation of the aggregated measure was applied when variance was increased for increased levels of measures. A pen within a room was the experimental unit, and nonsignificant block effects for room were excluded in the final model. Hock and navel scores were analyzed as binomial variables, being either zero or larger than zero (because only very few chickens had a score of 2). These binomial data were analyzed in a generalized linear model using a logit link. Day-old chick weights, organ weights, chick and intestinal lengths, and performance (mortality, FCR, water:feed ratio, European Production Efficiency Factor (**EPEF**) were analyzed using ANOVA with treatment as main effect. Measures that were performed on different ages (body weight, cleanliness, footpad dermatitis, hock burn injury and gait scores, intestinal weights, intestinal histology and pathology scores, and litter scores) were analyzed in a split plot model using ANOVA, with age within pen as residual term. In these split plot models, the interaction of treatment by age was tested. In case of 3 or more repeated measures (ages), an autoregression term was added to the model (when significant) to model the lag-dependent correlation of the repeated measure.

## Results

### Day-Old Chick Quality and Physical Development

The average percentage of nonhatched eggs at the hatchery (C eggs) was 4.25 and 2.30% for OH eggs. At D0 on average, 48% of the OH chicks had feed present in the crop. On-farm hatched chicks had a significantly higher, thus worse, score for navel and hocks at D0 as compared with C chicks. Predicted mean hock scores were 0.017 ± 0.012 for C and 0.167 ± 0.032 for OH, respectively (*P* = 0.006). Predicted mean navel scores were 0.175 ± 0.047 for C and 0.389 ± 0.054 for OH, respectively (*P* = 0.029).

At D0, body weight and yolk-free body mass of OH chicks was significantly higher as compared with C chicks (Table 1; F_1,6_ = 61.21, *P* < 0.001 and F_1,6_ = 50.61, *P* = 0.006 respectively). In addition, the relative gut and stomach weights were significantly higher for OH than for C chicks at D0 (F_1,6_ = 35.71, *P* = 0.009 and F_1,6_ = 97.27, *P* = 0.002 respectively), and OH chicks tended to have a higher liver weight than C chicks (F_1,6_ = 6.20; *P* = 0.089), but no differences were found in relative heart weight. Control chicks tended to be longer at D0 (Table 1; F_1,6_ = 3.86; *P* = 0.097) and tended to have a longer gut relative to BW than OH chicks (F_1,6_ = 4.72, *P* = 0.073), but no treatment differences were found for absolute gut and intestinal length and for intestinal length relative to BW (Table 1).

**Table 1:** Predicted means and least square differences (lsd) for body weight, yolk free body mass (YFBM), and residual yolk sac weight in grams (g), relative organ weights relative to YFBM (expressed in %) of heart, liver, stomach (gizzard plus proventriculus), and gut, chick length, and absolute and relative length (to body weight) of gut and intestines for control (hatchery-hatched, C) and on-farm hatched (OH) chicks at D0.

Indicator	C	OH	lsd	*P* value
Body weight (g)	41.79	47.12	1.67	**<0.001**
YFBM (g)	38.34	43.14	2.15	**0.006**
Residual yolk sac (g)	3.46	3.98	0.72	0.105
Relative organ weights (%)				
Heart	0.80	0.84	0.07	0.138
Liver	3.01	3.15	0.18	**0.089**
Gut	5.56	6.87	0.69	**0.009**
Gizzard plus proventriculus	7.18	8.44	0.40	**0.002**
Chick length (cm)	18.66	18.32	0.17	**0.097**
Gut length (cm)	49.10	51.5	4.46	0.237
Relative gut length to BW	1.18	1.10	0.09	**0.073**
Intestinal length (cm)	40.59	42.98	3.46	0.142
Ratio intestinal length: BW	0.98	0.90	0.10	0.111

Significant effects (*P* < 0.05) or tendencies (*P* = <0.10) are indicated in bold.

### Performance

A significant interaction between age and treatment was found for body weight development (Wald statistic = 26.68; *P* = 0.002) (Table 2). From D0 to D21, OH chickens were significantly heavier than C chickens (*P* < 0.05), whereas at D29, D32, and D40, body weights did not differ any more. Total first wk mortality tended to be higher in C pens than in OH pens (Table 3; F_1,6_ = 4.21, *P* = 0.086). Total mortality over the whole production period was significantly higher in C pens as compared with OH pens (Table 3; F_1,6_ = 8.33; *P* = 0.028). This was caused by a significantly higher percentage of chickens found dead in C pens than in OH pens (Table 3; F_1,6_ = 18.92; *P* = 0.005), as the percentage of culled chickens did not differ between the treatments (Table 3). Standard deviations of body weight at D40 were not significantly different. Although there was a tendency for a better FCR corrected to 1,500 g in OH pens than in C pens (Table 3; F_1,6_ = 4.74, *P* = 0.072), FCR corrected to 2,500 g and the net FCR did not differ between the treatments. The water:feed ratio did not differ significantly between treatments. The EPEF was significantly higher (better) for the OH than for the C treatment (Table 3; F_1,6_ = 7.50, *P* = 0.034).

**Table 2:** Body weight (back-transformed means ± SD) between D0 and D40 for control (C) and on-farm hatched (OH) chickens (in grams). Until D29, a sample of 50 chickens per pen was weighed. At D33, 280 thinned chickens were weighed per pen, and at D40, all remaining chickens per pen.

Age	Control	On-farm hatched	*P* value treatment
Day 0^1^	44.41 ± 3.30	48.79 ± 2.16	<0.05
Day 1	55.15 ± 1.38	59.30 ± 2.49	<0.05
Day 7	196.1 ± 6.8	209.5 ± 13.0	<0.05
Day 21	1,056 ± 26	1,090 ± 35	<0.05
Day 29	1,795 ± 60	1,834 ± 49	ns
Day 33	2,088 ± 48	2,138 ± 57	ns
Day 40	2,729 ± 55	2,792 ± 65	ns

1 Body weights of 50-day-old chicks per pen, measured 2 to 5 h after placement of C chicks. Note that these were different birds than the ones used for dissection (Table 1). The latter sample was measured on placement of the C chickens.

**Table 3:** Predicted means, least significant differences (lsd), and *P*-values for performance indicators of control (C) and on-farm hatched (OH) chickens.

Indicator	C	OH	lsd	*P* value
First wk mortality (%)	1.15	0.82	0.40	**0.086**
Total found dead (D0-40) (%)	2.93	2.24	0.93	**0.005**
Total culled (D0-40) (%)	1.07	1.16	0.20	0.319
Total mortality (D0-40) (%)	4.01	3.40	0.52	**0.028**
StDev of body weight D40^1^	339	344	17	0.554
FCR 1,500 g^2^	1.07	1.04	0.035	**0.072**
FCR 2,500 g^2^	1.48	1.46	0.024	0.110
Net FCR^2^	1.49	1.49	0.015	0.388
Water:feed ratio	1.80	1.82	0.06	0.352
EPEF^3^	430.3	445.1	13.25	**0.034**

Significant effects (*P* < 0.05) or tendencies (*P* < 0.10) are indicated in bold.

1 StDev: Standard deviation.

2 FCR: Feed conversion ratio, either corrected to 1,500 g (FCR1500; correction factor 0.01 per 25 g) or to 2,500 g (FCR2500; correction factor 0.01 per 50 g); net FCR is the FCR calculated over the whole production period between D0 and D40.

3 European Production Efficiency Factor (EPEF): {BW gain (g/D) × [100 − mortality (%)]}/(net feed conversion × 10).

### Litter Dry Matter Percentage and Litter Quality

**Figure 1:**
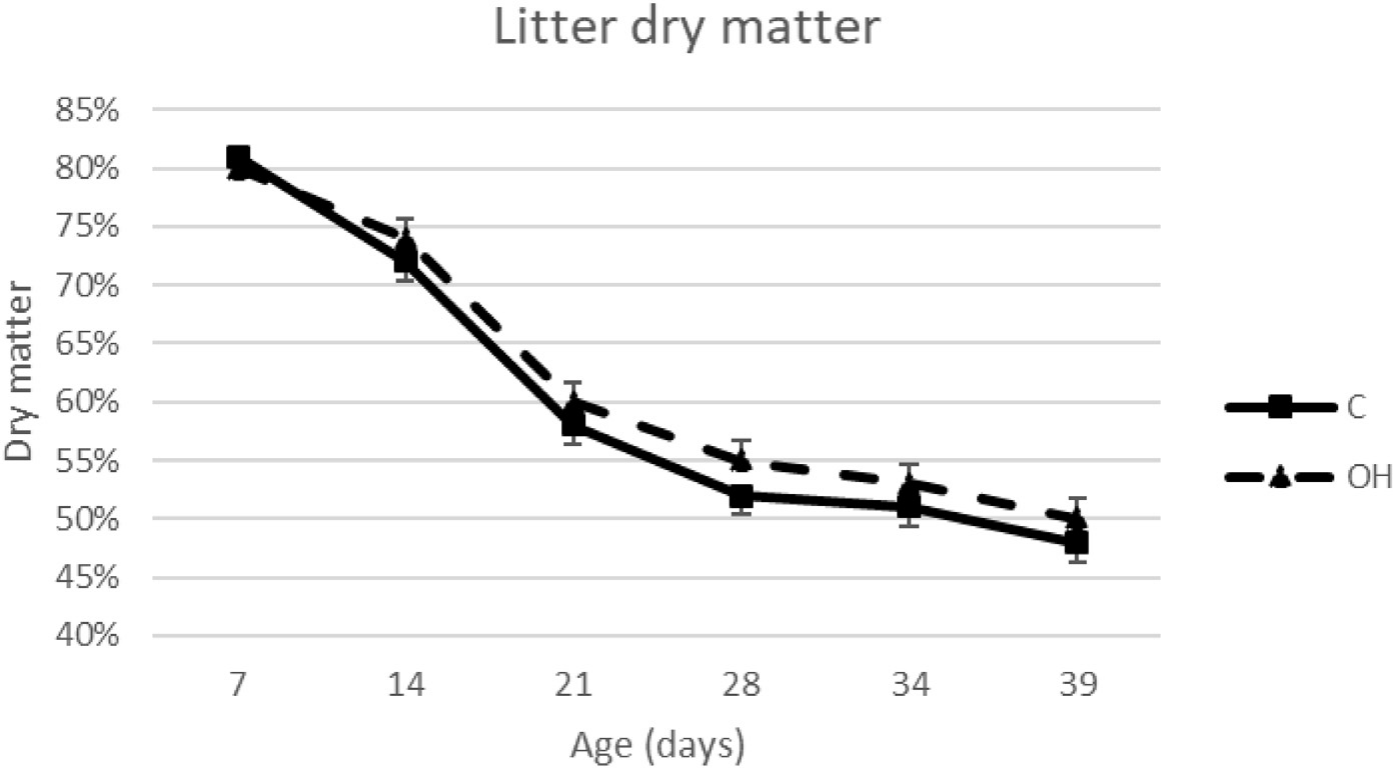
Predicted means and standard errors (se) of litter dry matter percentage for control (C) and on-farm hatched (OH) pens between 7 and 39 D of age. A significant effect of treatment was found (*P* < 0.001).

Figure 1 presents the litter dry matter percentages between 7 and 39 D of age. Litter dry matter percentage significantly decreased with age (Wald statistic = 1,123.49; *P* < 0.001) but was significantly higher for OH pens as compared with C pens (Wald statistic = 6.96; *P* < 0.05). No significant treatment differences were found for litter quality scores (Wald statistic = 2.92; *P* = 0.12), which were analyzed only with data from D14 onward, as scores for D4 and D8 were zero for all treatments. Litter quality scores significantly increased with age, except just after thinning (D35), after which the scores improved (Wald statistic = 767.6; *P* < 0.001). Mean scores per age for both treatments were as follows: D14: C 0.4, OH 0.2; d21: C 2.4, OH 2.0; D28: C 2.6, OH 2.2; D35: C 1.9, OH 1.6; and D39: C 2.4, OH 2.4.

### Gait, Footpad Dermatitis, Hock Burn, Cleanliness, and Injuries

Scores for gait, footpad dermatitis, hock burn, cleanliness, and injuries were all significantly increasing, that is getting worse, with increasing age (*P* ≤ 0.01 for all indicators). Only a tendency for a treatment effect was found for footpad dermatitis (F_1,6_ = 2.58; *P* = 0.09) with higher, that is worse scores for C chickens as compared with OH chickens (Table 4).

**Table 4:** Average scores (back transformed means) for footpad dermatitis (FPD), hock burn, cleanliness, injury, and gait at D21 and D35.

Indicator	Age (D)	C	OH	Treatment		Age	
				lsd	*P* value	lsd	*P* value
FPD	21	0.10	0.05	0.38	**0.09**	0.82	**<0.001**
	35	1.07	0.50				
Hock burn	21	0.05	0.05	0.52	0.49	0.36	**0.01**
	35	0.92	0.66				
Cleanliness	21	0.89	0.89	0.09	0.93	0.08	**<0.001**
	35	1.19	1.19				
Injuries	21	0.04	0.05	0.51	0.56	0.41	**<0.001**
	35	0.77	0.84				
Gait	21	1.95	1.99	0.03	0.16	0.04	**<0.001**
	35	2.45	2.50				

### Dissections and Intestinal Histology

Dysbacteriosis was scored at D28 and D39, and scores significantly increased with age (F _1,6_ = 30.12, *P* = 0.002) but did not differ between C and OH chickens (mean value of both ages combined: C: 3.01, OH: 2.67, least square differences [**lsd**]: 0.945, F_1,6_ = 0.74, *P* = 0.421). Likewise, coccidiosis scores (also scored at D28 and D39) increased with age (F_1,6_ = 27.58, *P* = 0.002) without a significant treatment effect (mean scores for both ages combined; C: 0.77, OH: 0.62, lsd 0.22, F_1,6_ = 2.85, *P* = 0.143). Femoral head necrosis scores significantly increased with age (F_1,6_ = 20.36, *P* = 0.004) and were higher for OH than C chickens at D39 but not at D28 (mean values, D28: C: 0.51, OH: 0.49; mean values D39: C: 0.75, OH: 1.03; lsd: 0.21, F_1,6_ = 15.68, *P* = 0.007). None of the dissected birds showed signs of TD. Gross pathology did not indicate any differences between both treatments (data not shown).

For total gut length relative to BW, a significant interaction of age∗treatment was found, with C chickens having longer guts relative to BW than OH chickens at D8, but not at other ages (Wald statistic = 12.03, *P* = 0.039; back transformed means for C: 0.584 and OH: 0.532 at D8). Cecal length relative to BW also showed an age∗treatment interaction (Wald statistic = 11.43, *P* = 0.041) with C having longer ceca than OH at D8 and D21 but not at other ages (back transformed means, D8: C: 0.078, OH: 0.070; D21: C: 0.235, OH: 0.223). No treatment differences were found for the ratio total intestinal length:BW and colon length:BW. Mean values for ratio intestinal length:BW were D8: C: 0.49, OH: 0.45; from D14 onward, averages were equal for C and OH and were: D14: 0.24; D21: 0.14; D28: 0.10; D39: 0.07 (treatment effect: Wald statistic = 3.23; *P* = 0.13). Mean values for ratio colon length:BW were equal for C and OH at all ages and were D8: 0.019; D14: 0.010; D21: 0.006; D28: 0.004; D39: 0.003 (treatment effect: Wald statistic = 2.08; *P* = 0.16). For villus length, crypt depth, and goblet cell characteristics measured only in the second cycle at D8, D14, and D21, only significant age effects but no treatment effects were found, and for villus:crypt ratio, no significant age or treatment effects were found. Mean villus:crypt ratios were D8: C: 4.05, OH: 4.14; D14: C: 3.86, OH: 4.01; D21: C: 3.45; OH: 3.49 (treatment effect: F_1,6_ = 0.48; *P* = 0.51; other data not shown).

## Discussion

In the present study, we showed that although OH chickens had a worse day-old chick quality, their body weights were higher until 21 D of age, and their total mortality until slaughter age was lower as compared with hatchery-hatched chickens, resulting in a significantly better European Production Efficiency Factor. With respect to welfare, we found a tendency for fewer footpad lesions and dryer litter in OH as compared with hatchery-hatched chickens. There were no indications of long-term differences in gut development between these treatments.

We previously studied the effect of on-farm vs. traditional hatching on commercial farms (de Jong et al., 2019). In that study, we found that effects of on-farm hatching on performance were mainly observed in the first wk, but not thereafter, and that there was a positive effect on broiler welfare resulting from reduced footpad dermatitis and a tendency for a better litter quality in OH as compared with hatchery-hatched flocks. We concluded that further study under more controlled conditions was required to exclude possible farm management effects. Here, OH and traditionally hatched chickens were compared in 3 subsequent production cycles under controlled conditions on an experimental farm, and results point into the same direction, that is improved welfare for OH as compared with hatchery hatched chickens but no higher body weight or improved FCR at slaughter age.

Analysis of day-old chick quality and development were in accordance with previous results (de Jong et al., 2019). Day-old OH chickens were heavier than hatchery-hatched (C) chickens, likely because of the fact that they could eat and drink immediately after hatching (Van de Ven et al., 2009). On average, 48% of the day-old OH chicks had feed present in the crop. This could reflect a variation in hatching time and thus in start of first feeding. On-farm hatching resulted in a higher gizzard and gut weight, likely because of being filled with feed. In the present study, we also find a tendency for a higher relative liver weight in the OH chicks. Some studies indicate that immediate posthatch feeding results in an accelerated development of digestive organs compared with chicks subjected to posthatch feed deprivation (e.g., van de Ven et al., 2013), which may explain the higher relative liver weight in OH as compared with C chicks, although not all studies found these effects (de Jong et al., 2017). Moreover, if effects of feeding on digestive organ development were present, these were only seen in the first days of life (see de Jong et al., 2017 for an overview of the literature). Intestinal development is stimulated by the intake of exogenous feed posthatch (Jin et al., 1998), and it is therefore suggested that immediate posthatch feeding results in longer intestines, although again not all studies found this effect (de Jong et al., 2017). In the present study, we even found a tendency for a higher relative gut length without differences in absolute gut and intestinal lengths in C vs. OH chickens at D0. The absence of differences in absolute gut length may have been caused by the variation in hatching moment and thus first feeding moment within the OH group, which may have resulted in a variation in physiological development of the chicks at D0. Especially, the early OH hatchers may have had an advantage in development because of early feeding, also as compared with the late hatchers (van de Ven et al., 2013), and on average, effects on absolute gut length could therefore be masked.

Day-old chick quality, as measured by navel and hock scores (Leksrisompong et al., 2007; van de Ven et al., 2012), was worse in OH compared with C chicks, confirming earlier results (de Jong et al., 2019). This could be because of suboptimal hatching conditions at the farm or to a less strict removal of second grade chicks in the OH groups (practiced by the animal caretakers) as compared with the C groups that were selected at the hatchery. Day-old chick length, often measured as an indicator of chick quality (Willemsen et al., 2008), also tended to be longer for C chicks. It is, however, interesting that the apparently worse day-old chick quality of OH as compared with C chicks did not result in higher first-wk or total mortality in the OH groups, which also confirms our previous results (de Jong et al., 2019). This further confirms the suggestion that day-old chick length does not predict later performance of broiler chickens (Willemsen et al., 2008) and raises doubt about the value of navel and hock quality as indicators of day-old chick quality in relation to mortality and performance in broiler chickens until slaughter age.

Body weights of OH chickens were significantly higher than C chickens until 21 D of age. Previous studies found a significantly higher body weight at day 7 but not at slaughter age in on-farm as compared with hatchery-hatched chickens (van de Ven et al., 2011; de Jong et al., 2019). Here, we measured body weight on a weekly basis during the whole rearing period and found that the higher body weight of OH as compared with C chickens lasted until 3 wk of age. A review of various studies under controlled conditions showed that immediate posthatch feeding significantly improves body weight at slaughter age compared with chickens that have been food deprived for 36 h posthatch or longer, but there is considerable variation between studies (de Jong et al., 2017). For example, Hollemans et al. (2018) observed a higher body weight only until 21 D of age in chickens that were immediately fed posthatch as compared with chickens that were posthatch feed deprived for 54 h. In the present study, the food deprivation period may not have exceeded the period necessary to find long-term effects, for instance because in the particular experiment the distance between hatchery and farm is short resulting in a transport time of only 45 min. Furthermore, effects were probably more significant for early than for mid and late hatchers in the OH groups (van de Ven et al., 2011; Lamot et al., 2014), resulting in an on average short-term effect of on-farm hatching on body weight. In line with these findings, net FCR at slaughter weight and FCR corrected at 2,500 g did not differ. FCR corrected at 2,500 g likely better represents the current situation regarding genetics and management as compared with the “old” FCR corrected to 1,500 g that more reflects the situation several years ago; thus, the trend for a better FCR corrected to 1,500 g in the OH chickens needs to be interpreted with care. Under controlled conditions, posthatch feed deprivation of more than 60 h resulted in impaired FCR at slaughter weight (de Jong et al., 2017), but this period of food deprivation was unlikely for most chicks in the C groups.

On-farm hatching had a significant and positive effect on the total mortality, which was not found in previous system comparisons (van de Ven et al., 2011; de Jong et al., 2019). This effect was because of a tendency for a lower mortality in OH groups in the first wk, as well as a lower number of chickens found dead between D7 and slaughter age in the OH as compared with the C groups. This suggests better health of OH chickens as compared with C chickens. Likely, early posthatch feeding contributed to this effect (de Jong et al., 2017). It cannot be excluded that other factors, such as chick handling (Knowles et al., 2004), dust, and pathogens in the hatchery (de Gouw et al., 2017) and stress because of transport as day-old chicks (Mitchell, 2009) in the C groups may also have contributed to the effect on mortality, but that merits further study as data are scarce. It is not clear why the positive effects of on-farm hatching on mortality were not found in previous studies, but perhaps individual farm management (de Jong and van Riel, 2020) or disease pressure may have played a role, reducing the differences between both hatching conditions. In the present experiment, all treatments were housed on 1 single farm and thus were exposed to similar management practices.

As regards the various welfare indicators that were included in the present study, we did not find significant differences between the treatments for gait, hock burn, cleanliness, and injuries. Only a tendency for fewer footpad lesions in OH compared with C groups was observed, which again confirms our previous study under commercial conditions (de Jong et al., 2019). These treatment differences in footpad lesions may be because of a lower litter moisture content in the OH as compared with the C pens (de Jong et al., 2014), although visual litter quality did not differ between the treatments. It is yet unclear how on-farm hatching affects litter moisture level. A possible explanation might be an improved gut development and/or health because of early feeding, resulting in better feces quality, but we were unable to demonstrate any differences in intestinal histology and gut lengths. However, the sample size in our study was relatively small. We only found that gut (D0, D8) and ceca (D8 and D21) length relative to body weight was longer in C than OH chickens, but effects were neither long-term (gut length) or consistent over ages (cecal length). The absence of differences in villus:crypt ratio may indicate that the absorption capacity of the jejunum does not differ between the treatments (Jin et al., 1998), which can thus not explain the difference in litter moisture content. Previous studies on effects of posthatch feed deprivation on intestinal development (weight, length, and histology) were nonconsistent, and if there were any effects of early feeding, these were usually only present at a young age (e.g., Gonzales et al., 2003; Maiorka et al., 2003; Mahmoud and Edens 2012; Lamot et al., 2014; de Jong et al., 2017). However, it remains possible that other effects that have not been included in the present study, such as differences in gut wall immunology or microbiome composition, may play a role in gut health and/or feces consistency, and this merits further study. Scores of dysbacteriosis and coccidiosis did not indicate any differences in gut health between the treatments. Although a difference in femoral head necrosis scores was found at D39 in favor of C as compared with OH chickens, no differences in gait score and TD were found, indicating that effects of on-farm hatching on leg health and locomotion were small or absent and not in favor of OH chickens.

In the present study, we performed a system comparison, meaning that various factors were different between the treatment groups. Therefore, we cannot specify which factors contributed to the differences in welfare indicators and initial body weight development between the OH and C chickens. It is very likely that the timing of the first feeding moment affects both performance and welfare (Willemsen et al., 2010; de Jong et al., 2017), but the effects of other factors such as day-old chick transport and environmental conditions during hatching on broiler performance and welfare are less clear. Hollemans et al. (2018) showed that day-old chick transport and early nutrition had interactive effects on fearfulness but that transport as such did not affect productivity in broiler chickens that were fed or feed deprived immediately posthatch. De Gouw et al. (2017) showed that both the hatching environment (presence or absence of dust and formaldehyde) and early feeding affected broiler chick development at D0, but that at D7, the effects of hatching environment on chicken development were absent. Whereas it is known that light during incubation affects fear and stress susceptibility of broilers at a later age (Archer and Mench, 2013; Archer, 2017), and it is unknown whether light during hatching (as opposed to darkness during hatching of C chicks in the hatchery) may have an effect on broiler welfare. Finally, it has been shown that commercial hatching routines may have a long-term effect on stress sensitivity in layers (Hedlund et al., 2019), but the effects on broiler chicken welfare and performance are unknown. Thus, the effects of these factors on broiler welfare and performance merit further study.

Our study was designed in such a way that OH and C pens were paired in climate controlled rooms to exclude a possible interaction with room effect when only 1 treatment was housed in a room. However, this design also required climatic settings to apply similarly to both treatment pens, and hence, it was not possible to adjust temperature settings to meet the specific requirements of each treatment. For example, the heavier OH chickens could have profited from a (somewhat) lower environmental temperature in the first wk. It remains to be studied in further trials how management can be best adjusted according to the requirements of early-fed, heavier chickens. Further, in the present study, the number of chickens was higher in the OH than C pens because of the fact that more chicks hatched than expected beforehand (especially in cycle 1 and 2) and the lower mortality in OH pens. This resulted in a somewhat higher stocking density in OH than C pens, and we cannot exclude that this could have had a (slightly) negative effect on some of the indicators measured, such as litter moisture content and quality and as a result on footpad dermatitis (Hall, 2001; Sanotra et al., 2002), although OH chickens still performed better with respect to these indicators than C chickens.

In conclusion, in the present study, we showed that on-farm hatching is beneficial for broiler welfare by reducing overall mortality and reducing litter moisture content and footpad lesion scores. This confirms and adds new information to previous comparisons between on-farm and traditional hatching (Van de Ven et al., 2009, 2011; de Jong et al., 2019). On-farm hatching does however not significantly improve body weight and feed conversion ratio at slaughter age, thus does not seem to have a long-term effect on productivity.
